# The Efficacy of Using Telehealth to Coach Parents of Children with Autism Spectrum Disorder on How to Use Naturalistic Teaching to Increase Mands, Tacts and Intraverbals

**DOI:** 10.1007/s10882-022-09859-4

**Published:** 2022-07-29

**Authors:** Jenny Ferguson, Katerina Dounavi, Emma A. Craig

**Affiliations:** School of Social Sciences, Education & Social Work, Queen’s University of Belfast, 69-71 University Street, Belfast, BT7 1HL Northern Ireland

**Keywords:** Autism, Telehealth, Parent training, Applied behaviour analysis, Verbal behaviour

## Abstract

There is a growing body of evidence supporting the use of telehealth to provide parent training in behaviour analytic interventions and researchers have begun to focus on international demonstrations of this model. The current study assessed the efficacy of a training package focused on naturalistic teaching strategies designed to upskill parents of children with autism spectrum disorder and provide them with ready to use strategies to increase social communication behaviours across verbal operants. Two parent–child dyads were trained to increase mand, tact and intraverbals during play. Parents displayed increases in fidelity for each strategy and viewed the training favourably. Both children showed gains across verbal operants, as captured by a multiple baseline across behaviours design.

**Supplementary Information:**

The online version contains supplementary material available at 10.1007/s10882-022-09859-4.

Difficulty in engaging in social communication is one of the core diagnostic criteria of autism spectrum disorders (ASD; American Psychiatric Association, 2013). This can manifest as difficulties in initiating social interactions, communicating wants and needs effectively and responding appropriately in conversation (American Psychiatric Association, 2013; Wetherby et al., 2007). Results from a large body of research have indicated that interventions based upon the principles of Applied Behaviour Analysis (ABA) are best placed to support individuals who have received a diagnosis of ASD (e.g., Hume et al., 2021; Makrygianni & Reed, 2010; Reichow et al., 2018; Wong et al., 2014); evidence of efficacy has included strategies shown to enhance social communication skills (e.g., Ingersoll et al., 2017; McGarry et al., 2019; Tanner & Dounavi, 2020). Furthermore, training parents in intervention procedures has demonstrable positive outcomes in terms of child communication and parental upskilling and empowerment (Antill, 2020; Degli Espinosa et al., 2020; Ferguson et al., 2022; Guomundsdottir et al., 2017); in fact, parent implemented intervention has accumulated enough of a research base to be considered as an evidence based practice (Hume et al., 2021).

Parents may take it upon themselves to avail of training courses which provide an overview of ABA theory and procedures. Online training is becoming increasingly popular and is widely available, including through commercial packages or University courses, often offered as initial training for gaining a professional credential in the field of ABA (e.g., to become a Registered Behaviour Technician; RBT^©^). Such training can provide parents with a cost effective yet comprehensive overview of ABA, for example, RBT^©^ training must provide 40 h of instruction based upon a broad task list of skills (Behavior Analyst Certification Board, 2020).

In the present study we recruited parents of children with a diagnosis of ASD who had completed a course meeting training requirements of a professional credential; this was either a course designed based on the RBT^©^ Task List or an Association for Behavior Analysis Verified Course Sequence (ABAI VCS) based on the BCBA^®^ Task List (Behavior Analyst Certification Board, 2017, 2018). Such parents have been excluded from prior research in this area. A commonality amongst the parents was that they undertook this training to advance their own parenting skills rather than to work in the field of behaviour analysis. Previous research on the use of online training packages has indicated that the inclusion of theory based training alone is not effective at increasing fidelity in the use of ABA-based strategies to clinically significant levels (Craig et al., 2021; Meadan et al., 2016). In order to reach meaningful levels of behaviour change, researchers have implemented a coaching component, where parents are taught to perform the steps necessary to teach new behaviour to their children (Heitzman-Powell et al., 2014; Meadan et al., 2016; Tanner & Dounavi, 2020). Without receiving coaching from an appropriately trained behaviour analyst (e.g., a BCBA^©^) parents may find themselves in the position of understanding ABA theory without having the practical skills to implement teaching based upon what they have learnt.

One solution would be for parents to utilise local expertise and gain coaching from professionals offering services within a commutable proximity. However, there is a dearth of appropriately trained behaviour analysts operating outside the USA. For example, Switzerland has a total of 22 BCBAs^©^ for an estimated total of 124,265 individuals with ASD and Germany has 51 BCBAs^©^ for an estimated total of 318,378 individuals with a diagnosis of ASD (Behavior Analyst Certification Board, 2022; Statista, 2020). It is clear from these numbers that utilising such expertise may not be an achievable or practical undertaking in terms of both availability and cost. For this reason, researchers have begun to focus on how to best support individuals who live in regions of the world where professional support is not readily available; telehealth has emerged as a viable platform for resolving this problem (Barkaia et al., 017; Craig et al., 2021; Craig et al., 2022; Neely et al., 2020; Tsami et al., 2019). Telehealth is the use of online communication technology to provide ongoing training, support and intervention in health related conditions (World Health Organization, 2021). There is a growing body of research indicating the efficacy of its use as a platform for providing parent training in ABA (Ferguson et al., 2019; Neely et al., 2021; Unholz-Bowden et al., 2020). The advantages of a telehealth platform have been further proved during the recent COVID-19 pandemic, which resulted in global lockdowns and closures of schools, universities, shops and other non-vital services. It also caused the cancellation of vital behaviour analytic services for individuals with ASD and has compelled service providers and funding bodies to search for appropriate alternative models of support, with many turning towards telehealth as the solution (Colombo et al., 2020; Rodriguez, 2020).

The present research adopted a telehealth training platform to offer parents of children with ASD training in naturalistic strategies designed to teach communication across three verbal operants: mands, tacts and intraverbals (Fryling, 2017; Sautter & LeBlanc, 2006; Skinner, 1957). Naturalistic teaching aims to create learning opportunities in child led activities across everyday environments. Researchers have provided training in naturalistic teaching via a telehealth platform to train both parents and professionals (Craig et al., 2021; Neely et al., 2019; Vismara et al., 2009; Wainer et al., 2017), with the majority of studies focusing on improving child communication skills (e.g., Meadan et al., 2016; Vismara et al., 2012, 2013, 2016). The decision to include and measure separate verbal operants is rare amongst previously published telehealth research, which has produced very few occasions of teaching any operant beyond mands. Tact and intraverbal responses have been taught using telehealth in direct teaching DTT sessions (Ferguson et al., 2020) and tacts have been discussed in research describing a transition of services to a telehealth model (e.g., Awasthi et al., 2021; Pollard et al., 2021). As it stands, no studies have measured intraverbal responses as a dependent variable targeted by naturalistic teaching delivered by parents and when research has included other operants (e.g., tacts) as a dependent variable, this has often been reported as part of an undifferentiated variable such as “functional verbal utterances” or “comments” (e.g., Ingersoll et al., 2016; Vismara et al., 2013, 2016). As tacts have not been reported in isolation, it is not possible to determine the isolated success of tact training from the amalgamated scores. Reporting increases in verbal operants separately will allow for a greater understanding of the success or difficulties in wider applications of a parent training program delivered via telehealth.

The current project also expands our knowledge by including parents located in different countries from that of the trainer and who came from different cultural backgrounds. Although research has demonstrated successful international training of professional interventionists (Barkaia et al., 2017; Craig et al., 2021; Neely et al., 2020), demonstrations of the effectiveness of international parent training are rare, especially in combination with naturalistic teaching. Many international demonstrations have included some face-to-face sessions in outpatient clinics (Gentile et al., 2022; Guðmundsdóttir et al., 2019; Guomundsdottir et al., 2017). Lee et al. (2022) used a telehealth platform to provide training in naturalistic teaching to parents residing in Mongolia. Researchers found promising gains in parental fidelity and provided a very useful framework for cultural adaptions but did not report on child outcomes. Most recently, Ferguson et al. (2022) provided a demonstration of the efficacy of using a telehealth platform to teach parents residing in the UK and Ireland social communication strategies. Results were consistent with US-based research as parents displayed increases in fidelity but variability in child communication outcomes was present (Ferguson et al., 2019; Neely et al., 2021). Child outcomes are extremely important as they are the end goal of any intervention. Research in telehealth has tended to focus on interventionist fidelity as a primary dependent variable, sometimes at the detriment to improvements in child behaviour (Neely et al., 2021). In the current study, although due to time restrictions parent fidelity was still considered the guiding dependent variable, training sessions continued beyond parental mastery, allowing children more time to practise.

Having taken place during the COVID-19 pandemic, this study provides an unprecedented and significant real-life demonstration of the application of telehealth during very difficult circumstances. Despite the cancellation of all other services for the children in the current project (e.g., schooling), the telehealth platform and home-based training enabled the continuation of much needed services by primary caregivers. Telehealth studies being conducted during the various global lockdowns have tended to focus on critical advice (e.g., Degli Espinosa et al., 2020), how to keep safe during the pandemic (e.g., Sivaraman et al., 2021) or have assessed the efficacy of switching to a telehealth model due to lockdown (e.g., Gevarter et al., 2022). The current study has provided a rare insight into the use of a telehealth platform during exceptional conditions, whilst aiming to maintain high research rigor throughout.

This project aims to build upon the current research base and answer the following research questions:

1. Can a telehealth model be used to train parents from diverse cultural backgrounds to use ABA-based natural environment teaching strategies to a high level of fidelity?
2. Do these interventions result in increased communication skills for the children across verbal operants?
3. Do these interventions result in increased levels of positive affect for the children?
4. How do parents rate the training they received in terms of social validity?
5. Can training and intervention effects be maintained overtime?

## Method

### Participants

#### Recruitment

One parent originally applied for a previous parent training project having seen a recruitment poster on a parenting social media post. They were deemed to be ineligible for the first project as they had prior training in ABA (Ferguson et al., 2022) but were subsequently invited to take part in the present study. The second parent was recruited through word of mouth, via advertisements on parent support groups and Facebook pages. Parents provided informed consent for their participation and on behalf of their child.

#### Eligibility

Parents were recruited for the project if they met the following criteria: a) be the primary caregiver of a child with a diagnosis of ASD, b) have access to a reliable internet connection capable of conducting video-calls, c) be willing to commit to at least 2 h per week of training and additional time to practise and provide consent to do so, d) have completed or be currently undertaking ‘official’ training in ABA, e.g., have completed RBT^©^ training or an ABAI VCS e) have no previous experience utilising ABA-based strategies professionally. Parents also had to be fluent in spoken and written English.

In order to be eligible, child participants had to: a) have a diagnosis of ASD as documented from an official diagnostic tool, such as The Autism Diagnostic Observation Schedule-2 (ADOS-2; Lord et al., 2012) or The Autism Diagnostic Interview-Revised (ADI-R; Rutter et al., 2003), b) be younger than 7 years at the time of recruitment, and c) present significant deficits in communication and social interaction identified through a pre-study behavioural assessment,(i.e. the VB-MAPP; Sundberg, 2008).

In total, three parent–child dyads were recruited for the project, however during the progression of the training, and due to a change in circumstances since recruitment it became apparent that a parent located in Pakistan no longer met eligibility. This was due to the parent changing employment to run an ABA service and taking part in a parallel parent training project offered by a University located in the United States. The parent followed through with the commitments of the project and the progression of the training, but her data were excluded from the study. Parent and child demographic information for the remaining two parents is presented below.

#### Rosa and Hugo Demographics

Rosa was a 32 years old female, native Spanish speaker originally from Latin America, who during the study resided in Switzerland with her son and husband. She had completed an undergraduate degree in law and was currently a stay-at-home mum. Rosa had completed RBT^©^ training and a Verified Course Sequence offered by a Spanish provider, which was based upon the BCBA^®^ Task List; this programme was offered to parents without the expectation of certification at the end and without the supervised practice component. Rosa reported that she focused only on the parts of the course useful to her son and did not take the final exam. Prior to the commencement of the study, Rosa completed a 20-question multiple choice test and scored 90%. Rosa’s first language was Spanish but she mostly communicated with her son in German and took part in the training in English.

Hugo was 3 years 4 months old at the start of the project. He had received a diagnosis of ASD confirmed via the ADOS-2 (Lord et al., 2012) when he was 2 years 3 months old. Hugo did not attend school or nursery during the course of the study but received 1 h of speech and language therapy per week. He was not receiving any ABA-based services during the study beyond working with his mum but had previously accessed 1 h of behavioural consultation per month for a period or 5 months, which ended 8 months prior to starting the project. During pre-study assessments, Hugo exhibited behaviour that was challenging, such as crying when demands were placed on him including during the pre-study assessment. Hugo displayed an ability to request for different preferred items and activities (e.g., ‘open’, ‘blow’, ‘swing’) although not spontaneously during play situations; these requests were demonstrated rarely during baseline videos. He could also tact preferred and common items when asked but not spontaneously and rarely in play situations. Hugo’s language was under echoic control but he struggled with correct pronunciation of words which was reflected in his scores on the Early Echoic Skills Assessment (EESA) included in the VB-MAPP. Hugo was also able to complete some simple intraverbal fill-ins such as some song lines but could not yet answer any questions or fill in information regarding feature, function or class of objects. Prior to commencing the project, Hugo’s teaching was delivered in the form of discrete trial training table-based activities. Rosa reported difficulty in moving away from these activities and lack of generalisation of Hugo’s language to more natural environments and novel stimuli.

All Rosa and Hugo’s sessions took place in their home in Switzerland. Hugo was present during all training sessions but would often join after a discussion of training strategies or the delivery of feedback. Prior to commencing the project, appropriate areas to conduct the coaching were identified. These areas had to contain preferred items and activities and have access to the internet to conduct the coaching sessions. Just after training commenced for the tacting strategy, Switzerland implemented a lockdown due to the COVID-19 outbreak. This meant that Hugo could not attend any of his scheduled appointments and could not leave his house other than to go for walks. Rosa used her own personal computer, webcam and wireless headphones for the project. The laptop was an Asus De A556U model and the webcam was a Logitech C920 HD Pro Webcam—Full HD 1080p. Rosa additionally used a mobile phone to film videos. All equipment was tested during the initial coaching sessions and although a tasks analysis of how to use the camera was available, this was not required.

#### Makena and Dennis Demographics

Makena was 38 years old upon the commencement of the study, she lived in Germany with her son and husband but was originally from East Africa. Makena was educated to college diploma level and had undertaken an RBT^®^ course. Prior to the commencement of the study, Makena completed a 20-question multiple choice test and scored 90%. Makena’s first language was English but she communicated with her son primarily in German and was a stay-at-home mother.

Dennis was 3 years 6 months old upon commencement of the project. He had a diagnosis of ASD confirmed via the ADI-R (Lord et al., 2012). He attended a mainstream kindergarten for 4 h per day and had never availed of any behaviour analytic services. Dennis’s language was under some echoic control and he scored positive on some targets of the EESA included in the VB-MAPP. During assessment sessions and baseline videos, Dennis displayed a very limited ability to request preferred items, with only two independent mands observed throughout the three baseline sessions. The first mand was an approximation of ‘balloon’ and the second was an approximation of “essen” meaning “eat”. Makena reported that Dennis was able to request for several other items by emitting one-word approximations, including “TV” and “iPad”, although these were not observed in the baseline videos. During all assessment sessions and videos Dennis displayed a very limited amount of eye contact and was only able to imitate one action when asked; he subsequently used this action as his response for all further imitation trials, despite other actions being modelled. Dennis could not label any objects when asked.

All Makena and Dennis’s sessions were conducted in her apartment located in Germany. Due to unavailability of childcare, Dennis was present throughout sessions, including when strategies were being discussed but during this time he was usually occupied with an activity, such as watching TV or using his iPad. Sessions took place in either Dennis’s bedroom or in the living room, both of which were set up with preferred items and activities. Makena used her own Acer Aspire E1-530 laptop, webcam and camera. She would also on occasion use her phone camera for recording videos or for conducting video calls as call quality was often better. Early on in the project, just as coaching had started, Germany introduced a lockdown due to the COVID-19 outbreak. This meant that Dennis could not carry out his usual schedule of activities and could not attend kindergarden; he also had to remain in his apartment at all times, apart from going out for walks.

### Training and Materials

#### Trainer

Training sessions were completed by the first author, a native English speaker, who had an undergraduate degree in Psychology, a postgraduate teaching qualification, Qualified Teacher Status (QTS) in England and a Master’s degree in Applied Behaviour Analysis. The trainer was a BCBA^©^ and was perusing a research PhD focusing on ABA. She had over 9 years’ experience delivering behaviour analytic interventions, including staff and parent training. The project was supervised by a BCBA-D who had nearly 20 years’ experience with ABA-based interventions and individuals with ASD and over 10 years of experience using telehealth. All training materials were produced by the first author and reviewed by the PhD supervisor. The trainer was located approximately 1,114 miles away from Rosa and Hugo and 965 miles away from Makena and Dennis and conducted training sessions in a University or private home office.

#### Materials

The trainer used a Lenovo ideapad 330 laptop and built-in webcam for all sessions. Prior to starting the coaching project parents completed a 20-point multiple choice knowledge test; they were then supplied with access to six training PDFs giving an overview of the theory behind ABA-based interventions. Both the test and the training documents were presented through Canvas, an online learning platform used to store training materials, tests and quizzes. Parents were then provided with four or five subsequent PDFs outlining the practical strategies they would introduce into the play sessions. These documents were emailed to the participants in turn, depending on their progression in the study. All coaching sessions were conducted via Skype video conferencing software and in accordance with the General Data Protection Regulation (GDPR), which is EU law protecting personal data. Skype in-call recording was used to record sessions. Videos recorded were uploaded by parents to WeTransfer, a GDPR compliant encrypted file sharing site.

### Research Design

The study adopted a single subject research design, specifically a concurrent multiple baseline design across behaviours. This experimental design has the ability to demonstrate whether there is a functional relation between an intervention and observed changes in target behaviours by systematically introducing the intervention to each behaviour in a staggered fashion, one at a time, whilst simultaneously keeping the other behaviours at baseline (Cooper et al., 2019; Dounavi & Dillenburger, 2013). In the current study, parent training was staggered to demonstrate control between the telehealth training received targeting specific parental and child skills in each training session and observed changes in both parental fidelity in using these strategies and corresponding child behaviour.

### Procedure

#### Baseline and Pre-study Assessment

A pre-study behavioural assessment was conducted prior to the start of the intervention. This included scoring questions taken from the Verbal Behavior Milestones and Assessment Placement Program (VB-MAPP) through parental interviews, observations, and live testing (Sundberg, 2008). This assessment was conducted over two Skype meetings for Dennis, taking 2 h in total, and three Skype meetings for Hugo taking 2.5 h. During this time parents were asked questions about their child’s abilities (e.g., “Does your child request for more than 6 different items?”) and were coached to directly test their child’s repertoire (e.g., blow some bubbles and pause and let’s see if he asks you for more or hold up the shoe, get him to look at it and say “what is this?”). The purpose of this assessment was to guide the target selection for each participant rather than provide a complete developmental assessment. Parents were also asked to submit three 12–15-min baseline videos depicting them playing and interacting with their child as they usually would to promote communication.

#### Coaching

Coaching commenced directly after the pre-study assessment and baseline measures were collected. Prior to starting the coaching sessions, parents were provided with written instructions on how to set up the play area, including incorporating preferred items, and how to set up the camera to ensure best view. Parents were coached to include four strategies in their play sessions. They were first taught to follow the lead of their child by gauging and building upon child motivation, using simple language, providing items to play with but following the lead of their child and not taking over the play (see Supplementary Table S1 for steps in the fidelity checklist). This was designed to pair play sessions with fun for the child and to allow the parents to explore and determine their child’s motivation. It also provided the opportunity to break any associations between play sessions and escape-maintained behaviour which had formed because of prior learning history, which is reflective of common practice in behaviour analytic services when starting a new program.

Parents were then systematically taught to include strategies to teach mands, tacts and then intraverbals into their play. Coaching in each strategy closely followed the expectations outlined in the fidelity checklists (Table 1). Positive feedback was provided when parents exhibited behaviour which closely reflected the fidelity checklist and suggestions were made to error correct any behaviour which differed. Each strategy was introduced progressively into the play session and parents continuing to include the previously mastered strategies in their play. At the end of each session, parents were provided with overall feedback and were asked if they had any questions or suggestions.

**Table 1:** Fidelity Checklist with Operational Definitions of each Strategy

Manding Strategy	Operational definition
1.Uses a motivation creation strategy	Uses a motivation creation such as: withholding items, providing small amount of item, adding on items to play. (Full descriptions can be found in Supplementary Table S2)
2.Utilises child initiation	Child demonstrating an interest in the toy or activity, to which access is currently controlled by the parent. Initiation can include reaching for toy, looking towards parent to gain more of something (bubbles, tickles etc.), pulling a parent’s hand towards an item or activity or vocalising a sound, word approximation or whole word requests. Parents should wait for this initiation before prompting. Score N/A if child is not motivated to gain access to anything under the control of the parent and negative if prompting takes place without this initiation or if this initiation is present but not utilised by creating a teaching moment
3. Eye contact	Parents should gain eye contact before prompting or providing access to item if mand was independent, but eye contact was not present. Prompting when necessary, as described in the strategy information sheet at correct prompt level. Prompts can include full sweep/search. Partial sweep/search and time delay. Score N/A if child is not motivated for an item or activity
4. Uses correct prompt technique (if applicable)	Uses prompting as described in the strategy information sheet (available on request). Prompts can include full echoic prompt, 3 and 5 s time delay. Prompts should only include the one target word and should be attempted three times before moving on. Score N/A if child is not motivated for an item or activity
5. Reinforces communication	Reinforcement should include praise (e.g. nice speaking, beautiful words!) and the item or activity if appropriate or a natural continuation of the activity. Items used to contrive motivation should not be provided unless communication has been observed or 3 unsuccessful attempts to prompt have been made. If item is provided after unsuccessful attempts, this should be of lesser magnitude or amount than if behaviour occurred
Tacting Strategy	Operational definition
1. Uses tact motivation creation strategy	Uses a motivation creation such as: hiding items, having items out of place, turning items over. (Full descriptions can be found in Table 2. Score negative if no tact strategy has been contrived
2.Checks child is paying attention to object/picture	Child demonstrates that they are attending to the target tact item by directing eye gaze towards the item and holding for at least a one second interval. Parents should not attempt to ask or prompt a tact if this attention is not present. Score N/A if no tact strategy has been contrived
3.Provides correct SD, if applicable	If running a impure tact trial parents should provide an appropriate SD in a clear voice. Appropriate SDs can include phrases like “What is it”, “What is that one?” “What is this one called?” and can be with or without a gesture to the item. Score N/A if child is independently demonstrating “pure” tacts or the parents is prompting impure tacts with just a gesture to the item. Also score N/A if no tact strategy has been contrived
4.Uses correct prompt technique or corrects error (if applicable)	Uses prompting as described in the strategy information sheet (in appendices). Prompts can include full echoic prompt, 3 and 5 s time delay. Prompts should only include the one target word and should be attempted three times before moving on. If child has made in error in the label, the correct label should be prompted using a full echoic prompt. Score N/A if no tact strategy has been contrived
5. Reinforces tact	Reinforcement should include smiling and lots of social praise (e.g. nice speaking, beautiful words!) and can also include a reference back to the tacted item (e.g., that’s a dog, yes it’s an elephant well done!). Score N/A if no tacting has occurred to be reinforced but negative if no or incorrect tact is reinforced
Intraverbal Strategy	
1.During appropriate play activity, pauses play and checks child is attending to them	Parent is engaging in an appropriate play activity conducive to intraverbals, (e.g., singing songs, playing games if chase, building a tower), pauses the play and checks child is attending to them. This can include, child shifting eye gage to the parent or turning their head towards them
2. Presents intraverbal	Presents intraverbal fill-in or question is a clear voice. Intraverbals will be individualised to child level. Score negative if no attempt at an intraverbal is contrived
3.Prompts answer (if applicable) and provides correct answer if error occurs	Uses prompting as described in the strategy information sheet (in appendices). Prompts can include full echoic prompt, 3 and 5 s time delay. Prompts should only include the one target word or phrase and should be attempted three times before moving on. If child has made in error in the label, the correct label should be prompted using a full echoic prompt. Score N/A if no attempt at an intraverbal is contrived
4. Reinforces (if applicable)	Reinforcement should include smiling and lots of social praise (e.g. nice speaking, beautiful words!) and can also include a reference back to the intraverbal (e.g., Yes! Dogs do say woof!). Score N/A if no attempt at an intraverbal is contrived but negative if an incorrect response or lack of response in reinforced

Manding Strategy adapted from “The impact of a telehealth platform on ABA-based parent training targeting social communication in children with autism spectrum disorder” by Ferguson et al., 2022, *Journal of Developmental and Physical Disabilities* (https://doi.org/10.1007/s10882-022-09839-8) Copyright^©^ 2022 Springer Nature

During mand training, parents were taught to use play scenarios which were designed to increase motivation in their children (Supplementary Table S2). During tact training parents were taught to create opportunities for spontaneous tacts to occur using the activities suggested (Table 2) and during intraverbal training they were taught to introduce intraverbals in the form of fun-fill ins (e.g., a lion says______) or simple Wh Questions (e.g., What do you do with pizza?) into their current play scenario. Building upon past research, the training utilised echoic prompts across all verbal operants (e.g., Coon & Miguel, 2012) and as per the fidelity check list parents were taught to attempt to prompt three time before moving on. Parents were taught to fade prompts using a time-delay method, after three successful echoic prompts were used, parents would insert a wait of 3 and then 5 s to provide an opportunity for the child to respond independently.

**Table 2:** Strategies taught to increase occurrences of tacting

Motivational strategy	Description	Examples
Hidden items	In this strategy you will provide your child with objects which are hidden within other objects or activities	Playing with a big tub of slime or sand you hide animals throughout the tub, which can be pulled out and labelled in turn You have items hidden under cups and turn each one over revealing the item and labelling as you go
Reveal	In this strategy you can hide the identity of pictures or objects by turning them over or revealing them dramatically and making a game of finding out what they are	During a puzzle place all the pieces on the flipped side. Reveal each in turn, with the aim that your child will label each as they put them in Spread various pictures across the floor and work with your child to turn them over and reveal what they are. This could also work as a ‘matching game’ to see if you can find the picture that matches the one in your hand Have a ‘special’ bag filled with items, make a big deal about what is in the bag before pulling out each item and labelling it with your child
Book or film	In this strategy you will use preferred books or films to increase spontaneous labels	Looking at a preferred book with your child start to label a few of the pictures on the page before gesturing to a subsequent picture and looking at your child while expect them to label it
Out of place	In this strategy you can place items in unusual places around the environment, where they would not usually be found. This is designed to increase attention to them and increase motivation to label them	You open the fridge and find toy vehicles in the there. As you pull them out, you label them with your child and laugh about how silly it is that they are in the fridge You stick pictures on the walls around the environment and play an ‘eye-spy’ game where you hunt down and label the pictures in turn
Sounds	It is not only things that can be seen that can be tacted but also things we can hear. Here you can play different sounds and teach your child to label them	Animal sounds game, play different animal sounds on your phone label each one with your child in turn

The first coaching session of each new strategy was split into two. The first half of the session involved a didactic overview of the verbal operant strategy, including the presentation of a PDF with a written rationale, a flow diagram describing in detail the expectations for each step of the strategy and a simplified version of the fidelity checklist showing expected behaviours. In the second half, parents were asked to set up an appropriate play situation and were coached to practise the strategies with their child. During the coaching sessions parents were first asked to identify the activity they had set up, although flexibility across the session to switch up play items based on child motivation was encouraged. Coaching then entailed the trainer watching the interactions between the parent and the child and providing necessary feedback via the in-ear headphones being worn by the parent. The trainer would provide behaviour specific verbal praise when the parent behaviour matched the steps of the fidelity checklist and the flow diagrams provided in the training documents list (e.g., “That’s excellent how you have provided the item after the request!” or “You have created excellent motivation here, well done!”). Parents were error corrected when they completed a step wrongly and suggestions were provided on how this could be improved (e.g., “You provided access to the item but did not prompt any language first; next time you can use the prompt prior to handing over the item.” or “You provided the start of the song lyric but did not wait long enough for Denis to fill-in the last word; next time wait at least 3 s to give him the opportunity to do this himself.”). Parents were also provided with suggestions for play scenarios that could be used during the sessions (e.g., “Why don’t you hide all the balls in the bag?”). The coaching ended with a general overview of what worked well in the session and what the parent should continue to work on more. (e.g., “There were some missed opportunities for including an intraverbal so let’s work on that.”). There was then an opportunity for the parent to provide feedback and to ask questions.

Subsequent coaching sessions involved spoken feedback of the most recent video submission, and further coached practice. After each coaching session, parents submitted a 12–15-min video depicting them playing with their child whilst incorporating all the strategies they have been taught so far. Coaching sessions took place once per week and had a scheduled duration of 1 h. There were occasions where the sessions were cut short by an unplanned parental appointment, connection issues or unscheduled nap times. There were also occasions when sessions exceeded 1 h in length due to a large number of parental questions or the need to support the parent to work through an episode of challenging behaviour. The average length of time of the coaching sessions was 48 min for Rosa and 46 min for Makena.

#### Maintenance

Parents submitted two further probe videos, after the completion of their final coaching session, with the aim to assess maintenance of gains in both child and parent target behaviours. These videos were collected monthly for approximately 2 months after the completion of coaching and were scored for all parent and all child dependent variables.

### Dependent Variables, Data Collection and Analysis

Videos sent by parents provided the main source from which data were analysed for all parent and child dependent variables. If possible, each video was standardised to disregard the first 2 min for reactivity and the remaining 10 min were scored. As the trainer did not speak German, a translator was used to translate the videos to English before these were scored.

#### Pre-study Assessment and Baseline Probes

Baseline videos were scored for all parent and child dependent variables and were used as further evidence of skills when completing the pre-study behavioural assessment. Parents’ knowledge of ABA theory was also tested at this stage by asking parents to complete a 20-point multiple choice test. Prior to completing the test parents were provided free access to didactic training materials, consisting of six PDFs covering ABA theory and six associated Prezi presentations used in a prior research project with the aim to offer them a theory recap resource that they could consult independently if they wished to (Ferguson et al., 2022).

#### Parent Dependent Variables

Dependent variables for the parents consisted of their fidelity in the use of the training strategies taught. Parental usage of each verbal operant strategy was measured using a fidelity checklist (Table 1). Prior to training in verbal operants, parents were taught to follow the lead of their child during play. A fidelity checklist for this can be found in Supplementary Table S1. Fidelity scores for this skill were collected to ensure parents’ mastery (e.g., 80% or higher on two consecutive sessions, not depicted in a graph).

Parents were then taught each verbal operant strategy progressively, i.e., each new strategy was only added if mastery had been demonstrated in the previous strategy. The mastery criterion was two consecutive sessions with 80% or higher fidelity score. Data were collected from a single video on all strategies. Despite parent behaviour being the main dependent variable that guided the progression of the study, the results of a previous research project had indicated that extending the length of time during which parents were coached into strategies may have beneficial results for child behaviour. As a result, parents took part in five coaching sessions for each strategy, regardless of whether they had already met mastery criterion in prior sessions.

Rosa’s first main target was her fidelity in using the manding strategy (see Table 1 for fidelity checklist and Supplementary Table S2 for an overview of motivation creation strategies taught for mands). Each step of this fidelity checklist was scored via partial interval recording for correct usage for every 20 s interval of the video. Scores were combined to form overall percentage for the strategy by dividing the number of intervals where the behaviour was observed by the overall number of intervals and multiplying by 100. Rosa was subsequently taught to increase tacts during play, using strategies designed to increase motivation in Hugo (Table 2). Tacts score was calculated in an identical fashion to the mand score (fidelity checklist for tacts can also been seen in Table 1). Rosa was then coached to introduce intraverbals into the play sessions. Depending on Hugo’s level of motivation in each session, intraverbals could be fun fill-ins involving actions or songs, which were often used at the beginning of the session along with manding or could be more complex intraverbals involving fill-ins or questions related to the function, feature or class of an object. Intraverbals were at first practised in context, e.g., whilst playing with pizza asking the question “Pizza is something you….?”, before moving onto extensions with out of context intraverbals, e.g., whilst playing with a toy duck first asking “What sounds does a duck make?” and then asking “What sound does a lion make?”, without a toy lion being present. The intraverbal fidelity checklist for this strategy can be found in Table 1, which was scored as per mands and tacts.

Makena was first coached on how to use strategies to increase mands and was subsequently taught how to increase fun intraverbal phrases and tacts during play. The same fidelity checklist (Table 1) and motivation strategies (Table 2 and Supplementary Table S2) were also used with Rosa.

#### Child Dependent Variables

##### Primary Child Behaviours

Child personalised communication targets were developed using results from the pre-assessment, baseline videos and discussions with parents. For both participants, prompted and independent occurrences of all three verbal operants occurring in each 10-min video were recorded and graphed independently. Three targets were chosen for each child. Hugo’s first target was to increase the frequency and independence of his mands during his play sessions, as well as to increase the variety of objects and activities he was asking for. His second target was to increase frequency and independence of tacts during play sessions, with a focus on increasing the occurrence of spontaneous tacts (as he already emitted some impure tacts when asked “What is this?”). His final target was to increase his intraverbal responses during play, including both intraverbal fill-ins and responding to questions regarding the features, functions and class of objects.

Dennis’s first target was to increase single word manding during his play sessions. His second target was to respond to simple and fun intraverbal fill-ins, such as “ready, steady, go” or “a dinosaur says roar”, or complete a known song. Dennis’s third target was to tact items in his environment. It was decided that intraverbals would be targeted before tacting for Dennis as his attending to stimuli around the environment was initially not conducive to learning to tact; additionally, his level of echoic control would make verbal prompting of tacts difficult without explicit motivation present. Therefore, intraverbals were targeted before tacts and were included in a very fun action filled way. As the study progressed, Dennis could attend more to the toys presented to him and his echoic repertoire greatly improved, which allowed tacts to be taught more effectively.

More specifically, mands were operationally defined as any attempt to request for the items or activity, which can include word approximation or whole word requests. Tacts were defined as any attempt from the child to label any item seen, heard or felt in their environment. If the child is reaching for, or engaging with an object after using the verbal language this should be considered a mand rather than a tact. Intraverbals are defined as any verbal response to a verbal stimulus, where the response and the stimulus differ. If a verbal response is emitted which is usually preceded by a verbal stimulus without the verbal stimulus being presented, then this is to be considered a mand or tact depending on the specific situation. The definitions aimed to provide a framework for differentiating between operants. However, the training also included “in-context” intraverbals, i.e., intraverbals which included questions or fill-ins which were directly related to the current play scenario, for example, when presented with a picture of a lion in a book the child may be asked “what does a lion say?”. Additionally, motivation may have been created when fun fill-ins such as “ready, steady, go” or “a dinosaur says roar!” where used. Considering both the in-context intraverbals and the fill-ins, it is important to note that there may be a distinction between pure intraverbal responses, i.e., responses solely evoked by a verbal discriminative stimulus, and multiply controlled intraverbal responses, which may follow verbal and other antecedent variables that combine to bring about the response. For example, a motivating operation might be created in the fun fill-ins or a visual stimulus can be present together with a verbal antecedent in the in-context questions (Palmer, 2016). Despite the frequent presence of multiply controlled intraverbal responses in natural settings and the fact that these are examples of how early intraverbals are often taught (DeSouza et al., 2017), for the current study, examples of multiply controlled or impure intraverbals were classified as intraverbals for the purpose of data collection, as long as the primary controlling variable was a verbal discriminative stimulus that sometimes but not always co-existed with an additional antecedent.

##### Child Affect

In addition to the primary child dependent variables, data were also collected on the occurrence of positive child affect for both participants using partial interval recording with intervals of 20 s. For data collection, the following definition of child affect was used: “at least one instance of smiling (upward curve of the mouth, with or without showing teeth) and/or laughing during interval, not to coincide with any occurrences of behaviour that challenges”.

### Data Analysis

Parental fidelity in their use of each strategy was graphed and examined for changes in level, variability and trend. Tau-U was used to calculate effect sizes of changes in parent fidelity the combined strategies for each parents. Scores for all child variables were graphed and visually analysed for changes in level, trend and variability. In addition, Tau-U, a statistical model capable of determining effect size in single subject research by examining the percentage of non-overlap in data between conditions was used to analysis effect sizes for the changes in scores for each child behaviour, for the combination of all three behaviours for each child and for both participants’ data combined (Parker et al., 2011). Effect sizes can be rated as weak (< 0.66), medium (0.66–0.92) or large (> 0.92) (Parker & Vannest, 2009; Rispoli et al., 2013).

### Social Validity

Social validity data were collected through a questionnaire and an interview assessing how the parents viewed the project. The questionnaire was an adaption of the behaviour intervention rating scale (Elliott & Treuting, 1991) and was scored using a 5-point Likert scale (1 = strongly disagree, 2 = disagree, 3 = neutral, 4 = agree and 5 = strongly agree), with the inclusion of project specific questions relating to strategies taught and the telehealth platform (Table 4). with the questionnaire included a space in which to provide written feedback if parents wished to. A semi-structured interview was then conducted, which consisted of seven open-ended questions (e.g., “What would you change about the project?”; full set of questions available upon request).

### Interobserver Agreement (IOA)

A second rater scored at least 33% of videos across all dependent variables and experimental conditions; the rater was blind to which phase of the intervention the video corresponded to. Scores of parental fidelity and child affect were computed using trial-by-trial IOA and main child variables utilised total count IOA. IOA was 92.5% (range: 81–100%) for parental fidelity scores, 92.5% (range: 79–100%) for child mands, 94.5.5% for tacts (range: 83–100%), 96.5% (range: 80–100%) for intraverbals and 89.5% (range: 79–97%) for affect. Scores can be found in Table 3.

**Table 3:** IOA calculations for parent and child scores

Parent	Rosa	Makena
Mand Strategy % *M*	94	92
*Range*	87–98	86–97
Tact Strategy % *M*	95	85
*Range*	83–100	81–100
IV Strategy % *M*	96	93
*Range*	81–100	83–100
Child	Hugo	Dennis
Manding % *M*	91	94
*Range*	79–100	89–100
Tact % *M*	94	95
*Range*	87–100	83–100
Intraverbal % *M* *Range*	97 82–100	96 80–100
Affect% *M*	85	95
*Range*	79–88	89–97

## Results

### Parent Main Variables

#### Rosa

**Fig. 1:**
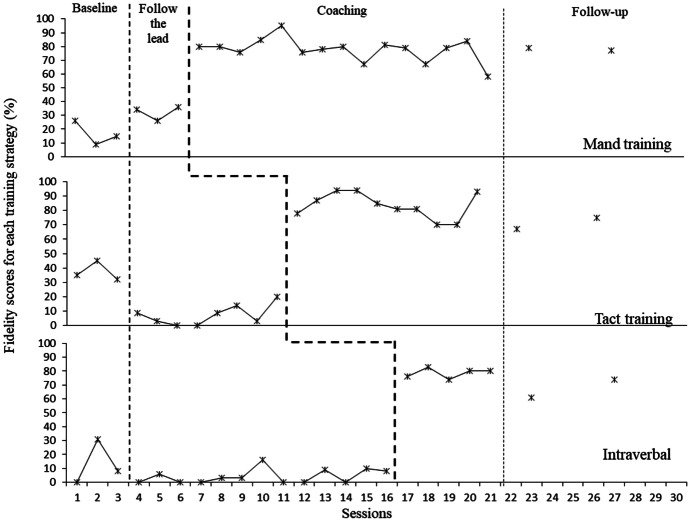
Rosa’s fidelity at using mand, tact and intraverbal strategies during play sessions

Figure 1 shows Rosa’s fidelity scores across the 10-min videos for each training strategy taught. Rosa’s baseline levels of fidelity in using each of the training strategies into her play sessions were for the most part low and stable. When Rosa was introduced to “following the lead” of her child, there was a small rise in mand training fidelity and a small reduction in the fidelity of both tact and intraverbal training. In addition, and although not shown in graphs, Rosa’s fidelity of implementing the follow the lead strategy was measured to ensure scores reached mastery before moving on with specific verbal operant training and Rosa reached mastery by the third session.

When Rosa was introduced to a specific training strategy there was a subsequent rise in her fidelity scores for that strategy (e.g., her fidelity in manding increased substantially to 80% in the first two sessions upon initiating training in that strategy reaching mastery; similarly, her intraverbal scores increased to 76% and 83% upon the start of training for that strategy having displayed no prior increase). Observed increases in fidelity were for the most part sustained, however there was a slight increase in variability upon introduction of new strategies. These data are suggestive of a functional relation between the training provided and increases in Rosa’s fidelity. To further support this conclusion, the Tau-U test was used to provide a combined effect size for the data set as a whole. This test indicated a strong effect size with Tau-U = 0.1 (z = 4.23, p < 0.001 with 95% CI [0.54,1]), providing additional support for the presence of a functional relation, as no overlapping data were present across experimental conditions of the whole dataset for Rosa.

#### Makena

**Fig. 2:**
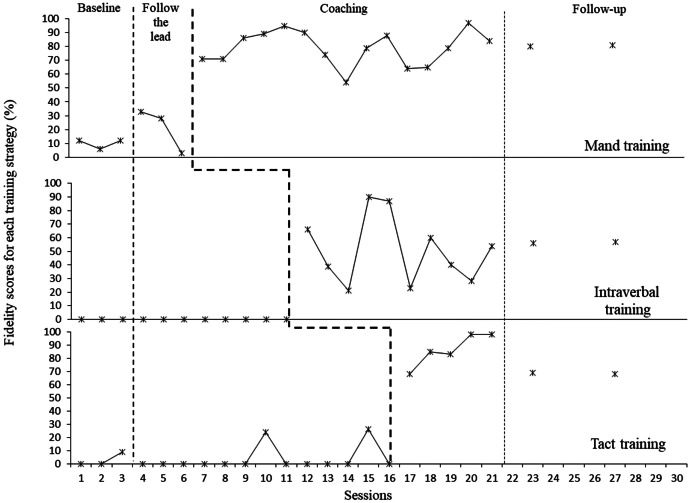
Makena’s fidelity at using mand, tact and intraverbal training strategies during play sessions

Makena’s baseline levels of fidelity in using training strategies were low and stable (Fig. 2). Makena was then introduced to “following the lead” of her child, after which there was a small rise in mand training fidelity for the first few sessions. Makena was adopting the manding strategy, whilst her fidelity in using tact and intraverbal strategies remained at zero. In addition, and not shown on the figure, the fidelity of Makena’s use of implementing the “follow the lead” reached mastery by the third session. After Makena was introduced to each strategy there was a corresponding increase in her fidelity scores (e.g., her scores using the manding strategy increased to 70% after one coaching session) indicating a functional relation was present. Changes in Makena’s scores were sustained at levels higher than baseline with manding and tacting fidelity scores remaining around mastery level. There was some variability in her intraverbal fidelity scores. Additionally, the Tau-U test was used to provide a combined, omnibus effect size for the data set as a whole indicating a strong effect size (Tau-U = 0.96, z = 4.09, p < 0.001 with 95% CI [0.50,1]), with no overlapping data at any point across all three experimental conditions.

### Child Main Variables

**Fig. 3:**
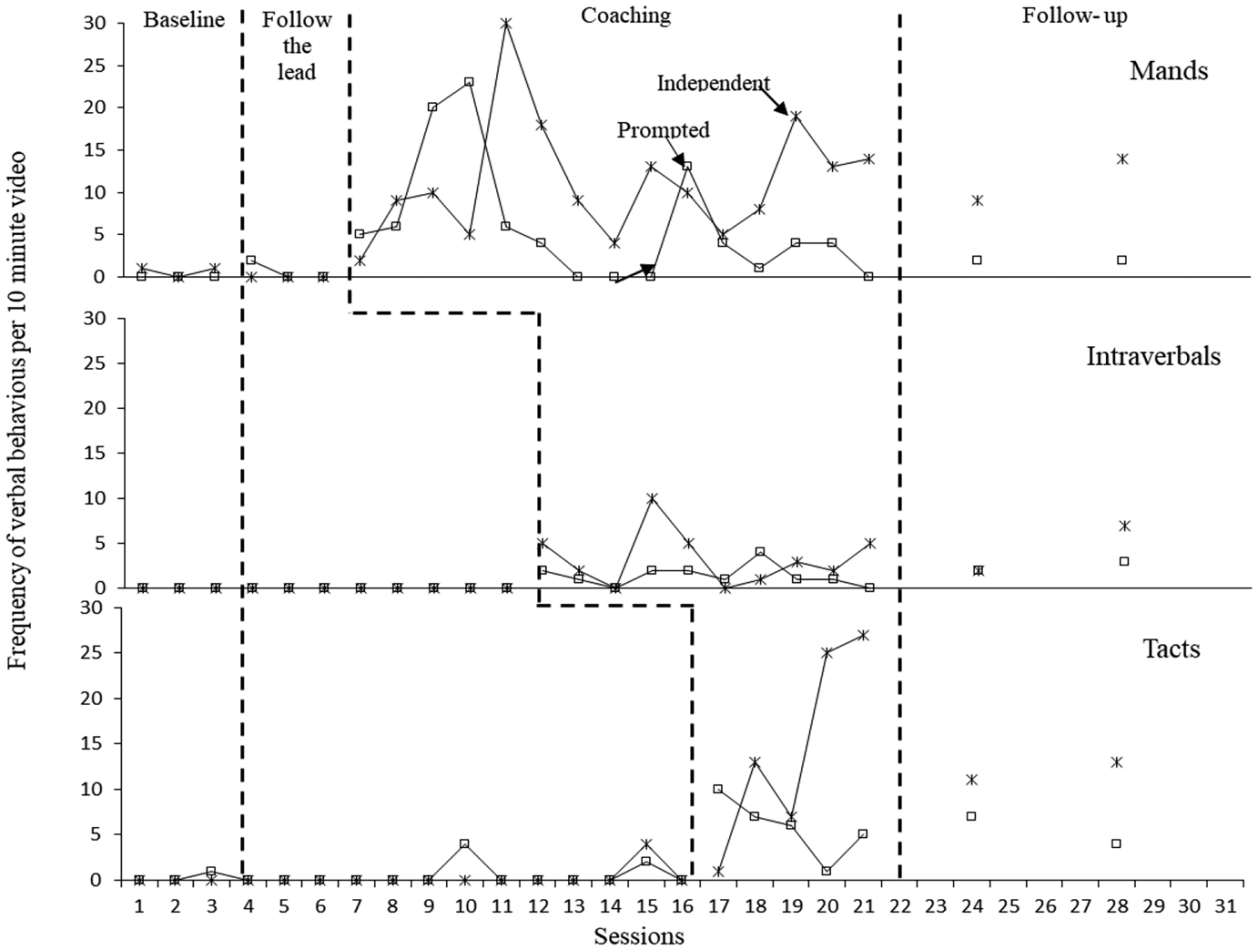
Hugo’s frequency of mands, tacts and intraverbal responses per 10-min video

**Fig. 4:**
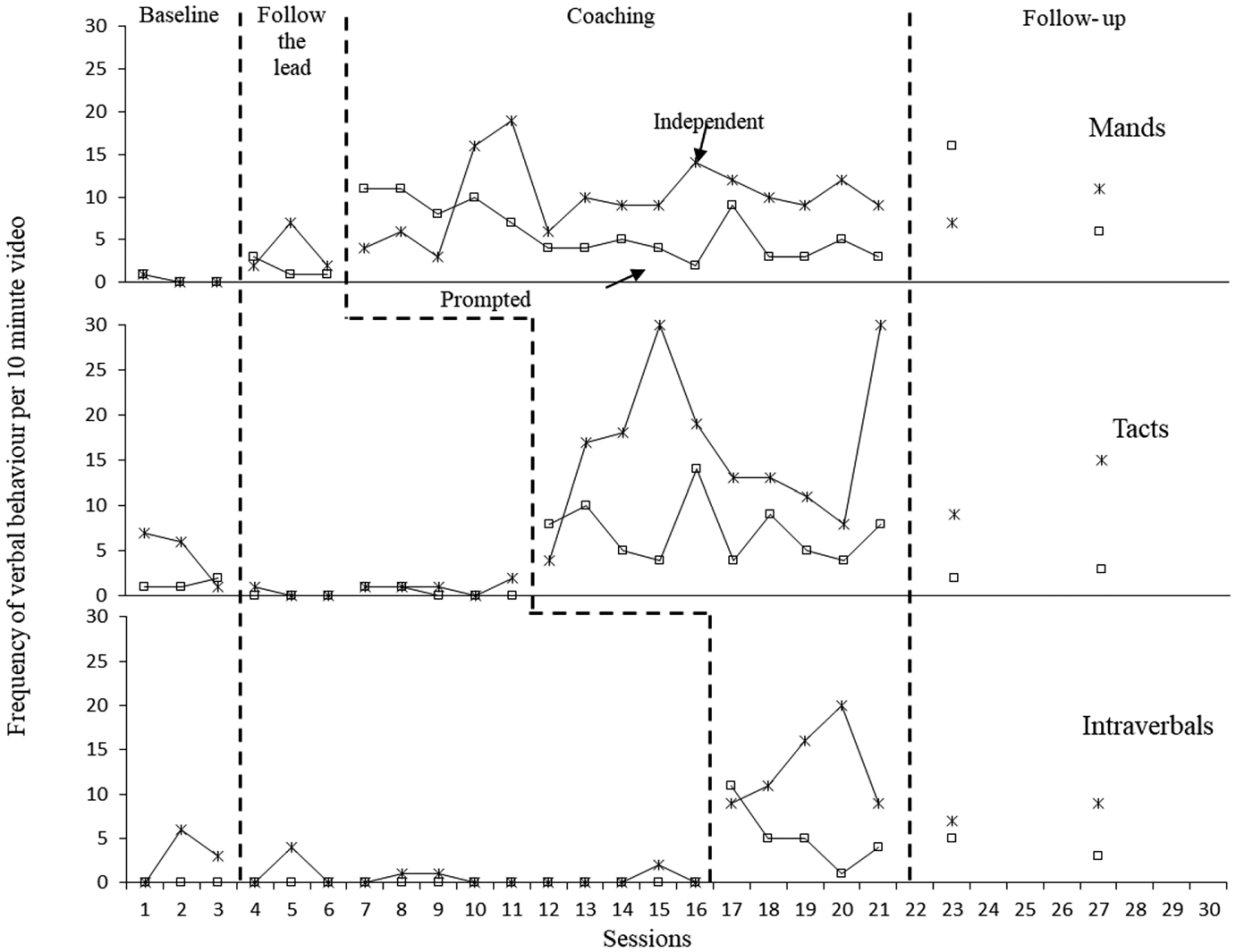
Dennis’ frequency of mands, tacts and intraverbal responses per 10-min video

Figure 3 shows the frequency in Hugo’s use of each verbal operant across each 10-min session. Figure 4 displays Dennis’ use of each verbal operant. Both Hugo and Dennis displayed low and stable baseline levels in all three verbal operants, with Hugo displaying a decreasing trend for tacts. The introduction of parent coaching on how to increase each verbal operant in play sessions was directly followed by an increase in frequency in using the corresponding operant for both Hugo and Dennis. This serves as proof of a functional relation between coaching and changes observed in Hugo’s use of language, which was mediated by changes observed in parental behaviour, i.e., fidelity of implementation of specific strategies. During Hugo’s manding sessions this was initially observed as an increase in prompted mands followed by an increase in independent mands, likewise with Dennis’ tact scores. In addition to the data displayed in figures, data were also collected on whether Hugo tacts followed a supplementary verbal instruction from Rosa such as “What is it?” or whether they were pure/spontaneous. During baseline there were only four occasions of spontaneous tacts across all sessions. After the implementation of direct coaching in tact training, Hugo used pure tacts more frequently (*M* = 11.7, 77%, Range: 3–28) than impure tacts (*M* = 4.5, 33%, Range: 1–10), indicating a large increase in the spontaneity of his tacting. As with parents’ data, there was some variability in frequency when new strategies were introduced. This was observed most strongly in Dennis’ intraverbal scores.

In addition to visual analysis, effect sizes were calculated for combined scores across verbal operants. Strong effect sizes were identified for Hugo’s increases (Tau-U = 0.96, z = 4.09, p < 0.001 with 95% CI [0.50,1]). Likewise, Dennis’ scores indicated a strong combined weighted effect size for the data taken on independent verbal behaviour across all three verbal operants (Tau-U = 0.93, z = 3.97, p < 0.001 with 95% CI [0.47,1]).

### Positive Affect

**Fig. 5:**
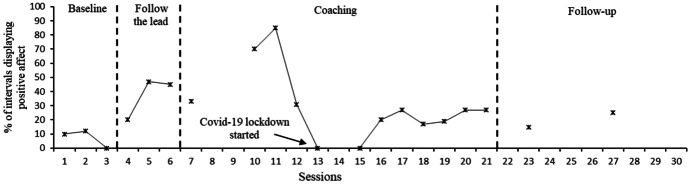
Percentage of intervals in which Hugo displayed positive affect. *Note. Mand training commenced in session 7, tact training in session 12 and intraverbal training in session 17

**Fig. 6:**
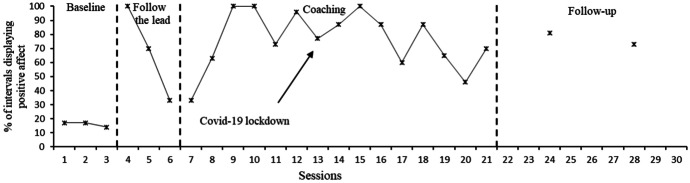
Percentage of intervals in which Dennis displayed positive affect. *Note. Mand training commenced in session 7, tact training in session 12 and intraverbal training in session 17

Figures 5 and 6 display the percentage of intervals in which positive affect was displayed by Hugo and Dennis respectively. Missing data were present in Hugo’s sessions 8, 9 and 14. This was due to an obscured camera angle being used, which resulted in an inability to clearly see affect for the majority of the video (e.g., filming from above whilst Hugo focused on toys in front of him). Hugo’s data indicated a rise in positive affect displayed during the “Follow the lead” and manding sessions but this returned to near baseline levels for the remainder of the sessions. Upon commencement of the coaching component, there was an upward trend in Dennis’ positive affect scores, which stabilised at this increased trend after the third coaching session; scores remained significantly higher than at baseline sessions for the whole of the coaching sessions.

### Social Validity

Both Rosa and Makena filled in the same 10-question social validity questionnaire used in a previous study (Ferguson et al., 2022) using a 5-point Likert scale (1 = strongly disagree, 2 = disagree, 3 = neutral, 4 = agree and 5 = strongly agree); specific questions and responses can be found in Table 4. Both parents rated the project very highly, they found the platform acceptable, the strategies easy to implement in their current play and the outcomes for their children favourable. In addition to the questionnaire, both parents completed an open-ended interview and were invited to provide any additional feedback on a written form. Both parents once again reiterated their preference for this kind of play-based intervention and felt the motivational activities worked well for their children; they both felt their children had benefited greatly from taking part in the project. Rosa commented “*After finishing the programme, I feel more secure to play with my son, I've learned that ABA can be fun too and my son is happier that we use the naturalistic teaching*.” This statement indicates that her confidence improved as a result of taking part in the project and her son enjoyed the sessions.

**Table 4:** Results of Social Validity questionnaire

Question	Rosa	Makena
1. I found the format of the telehealth training platform acceptable	5	5
2. The technology was easy to use	5	5
3. The timeline needed to complete this training was acceptable and manageable	4	5
4. I understood the theory and strategies taught in this training	5	5
5. The training provided me with strategies to interact with my child more effectively	5	5
6. The training increased my confidence as a parent	5	5
7. I feel like my child enjoyed taking part in the play sessions	5	4
8. I enjoyed taking part in the play sessions	4	5
9. My child showed some improvement in communication skills	5	5
10. I would recommend this training to others	5	5

1 = strongly disagree, 2 = disagree, 3 = neutral, 4 = agree and 5 = strongly agree

## Discussion

The present study was conducted with the aim to determine if a telehealth platform could be used to successfully deliver parent training in naturalistic strategies designed to increase child communication across multiple verbal operants. We focused on parents who had already completed theoretical training in ABA but lacked practical experience and who were from a different cultural background from the trainer and living in a different country. Findings indicated parents could be taught to a high level of fidelity to include training in multiple verbal operants during their play sessions with their child, resulting in a marked increase in child communication targets. This is a rare addition to the telehealth literature which has thus far primarily included teaching mands in isolation (e.g., Neely et al., 2016) or teaching less specific dependent variables such as “functional verbal utterances” or “social initiation” targets where operants are combined (e.g., Meadan et al., 2016; Vismara et al., 2013). No prior study has specifically targeted intraverbals in naturalistic teaching. This provides a demonstration of an extension of the telehealth platform, not previously seen.

In the current study both parents showed an increased fidelity in using each strategy only after live coaching initiated reaching mastery for each strategy during coaching sessions. Both geographical and cultural differences were overcome and did not pose any difficulties to the progression of the study, the outcomes observed, or the positive feedback provided upon completion. Because both parents spoke good English, the use of a German translator was only required for scoring videos. Additionally, the second author had extensive experience providing consultation to families from a range of countries including both countries represented in the project, therefore safeguarding cultural sensitivity. Prior research has shown translators can be used to facilitate coaching sessions whilst also providing information about specific cultural norms (Tsami et al., 2019). Whilst this may have had a limited benefit in the current project as both participants were originally from different countries and cultural backgrounds from their country of residence, it may have had a benefit in confirming the intelligibility of the words spoken by the children. As the trainer spoke no German it was not possible during the sessions to advise the parents of best approximations for shaping articulation or pronunciation. This was left to the parents to determine, who could not always provide the objective ear required. Having a translator during the sessions would have been useful to determine articulation and pronunciation as understood by non-family members. Focusing on parents who had already sought out theoretical training in ABA is a useful addition to the literature. This model may in fact already be commonplace in real life settings. It could also have practical implications as BCBAs^©^ could suggest parents avail of a low cost or free training course prior to starting to offer parent coaching in specific strategies, saving time and money and maximising intervention efficiency.

The second aim of the study was to assess the effect of the parent training on child communication outcomes. The study extended the number of coaching sessions the children took part in to rectify limitations identified in the past literature (Ferguson et al., 2022). Therefore, five training sessions in each verbal operant were completed for each dyad. This was regardless of whether the parents had already reached the mastery criterion during prior sessions. Guaranteeing five data points in each condition also ensured that the study meets without reservations the What Works Clearing House standards for single subject research designs (What Works Clearning House, 2020), ensuring high rigour throughout.

Both children displayed successive increases in their use of each verbal operant, only when parents were coached to teach specific operants in their play. Within the tact category, Hugo’s use of spontaneous or “pure” tacts vs impure tacts was measured showing a great increase as the study progressed. The strategies taught were designed to increase child motivation to tact and share their environment with others. Hugo’s ability to do so appears to have increased. Motivation to engage in social communication has been recognised as a pivotal skill, linked to thousands of additional behaviours (e.g., Lei & Ventola, 2017; McGarry et al., 2019; Mohammadzaheri et al., 2014). Acquisition of this skill will hopefully enable Hugo to reach best outcomes in the social communication domain as a whole. Although out of the scope of the current project, future research could focus on longer term outcomes.

Both parents rated the project extremely favourably, they were happy with the telehealth platform and the changes observed in their children. We also collected data on child positive affect. Positive affect measures can be used as behavioural indicators of private events of “happiness” (Ramey et al., 2019), providing a potential indicator of child social validity. There was a large increase in positive affect for Dennis that maintained throughout the sessions. Hugo’s data were more variable. There were limitations in these measures with several instances of missing data due to obscured view of Hugo’s face. This could be rectified in future research by clarifying with parents the importance of showing the child’s face throughout the sessions, as was done with Dennis, to ensure eye gaze data could also be collected. One potential explanation for the variability observed in Hugo is that the study utilised “neurotypical” indices of happiness, which may not be relevant to all individuals with ASD. When Hugo was completing an apparent preferred activity, he would often engage in a large amount of self-stimulatory behaviour, such as non-word vocalisations or flapping his hands. These were not captured in the current data collection system as positive affect was defined in a neurotypical manner. Although it is beyond the scope of the current project to assess if these behaviours were indeed indices of happiness for Hugo, future research could include more individualised measures of happiness to ensure the validity of the behaviour being measured.

This study was completed under unprecedented conditions in the spring/summer of 2020, just as the COVID-19 pandemic swept across the globe. Both families and the trainer were mandated to enter a period of lockdown. This happened after session 13 for Rosa/Hugo and session 12 for Makena/Dennis. For both families this remained until the end of the project and all other services were placed on hold. This had a bigger impact on Dennis who had been attending a mainstream kindergarten for 4 h per day, but Hugo also had speech and language sessions cancelled and both children could only leave the house once per day for walks or a visit to the local park. Due to the nature of telehealth, training was able to continue with minimal, if any, impact on the progress of the study. However, it is possible that there could have been unforeseen effects of child satiation to reinforcers, which has been identified as a potential problem due to unlimited access to reinforcers during a lockdown period (Degli Espinosa et al., 2020). It is hoped that this effect was mitigated by the naturalistic and motivational nature of the study. Parents were taught to add value to the play environment and the toys in it. When toys were available outside of play sessions, this value would not be as great, thus motivation was still present to play with the parent and toys during the sessions. Feedback from parents taken during the height of the pandemic in Italy has suggested that the use of NT strategies to create motivation and teaching opportunities would have been too effortful during the extreme circumstances of lockdown (Degli Espinosa et al., 2020). This was not the case for the current study but the parents were in a unique position as they had already been implementing these strategies prior to lockdown commencing. They also had strong motivation to learn about ABA and had sought out training in this area prior to the study commencing. Whether this could generalise to a wider range of parents under similar circumstances remains to be seen. The successful completion of the study demonstrates the utility of a telehealth model even under such unforeseen circumstances.

There were several limitations to the study. The main one being the ceiling effects of the data collection system and the feasibility of parents including all three strategies into a single 10-min play session video. It was not deemed a reasonable expectation to collect three separate videos, one for each verbal operant, as this would have resulted in the collection of 63 videos across the study, placing an unnecessary strain on already overstretched parents. Therefore, parents were asked to send one video which would include examples of all three training strategies when they were introduced in turn. This may have led to a ceiling effect due to time limitations, where the parent simply did not have enough time to fit in all strategies to each video, resulting in variability in both the parent fidelity data and the child outcomes. The data collection system used in the study captured occurrence of each strategy while simultaneously measuring fidelity of implementation. Videos displaying few occurrences of parental use of a particular strategy were evident throughout the study, especially when a new strategy had just been introduced. This was evident on a small scale in Rosa and Hugo’s data (e.g., Rosa’s use of the tact strategy dropped slightly when intraverbals were introduced, which had a knock-on effect on the number of tacts displayed by Hugo) but there seemed to be a larger variance in Makena’s scores. For example, Makena’s use of the intraverbal strategy reached mastery after the fifth coaching session. In the next session, when the tacting strategy was introduced, her scores dropped back to between 20–60% and remained in this range for the rest of the sessions. Further analysis of her scores indicated that there was a reduction in the occurrence of her using the intraverbal strategies rather than a reduction in fidelity of implementation. This could be explained by a few factors. Although play activities were child-led throughout, after the introduction of tact training more focus was placed on activities with toys to tact as opposed to social play, such as playing chase or singing songs with actions. For this reason, Makena may have benefited from fluency-based training of each strategy. Fluent responding is accurate and quick, without hesitation (Weiss, 2001) and can have benefits in the retention and application of skills in new activities (Binder, 1996) (i.e., the ability to maintain and generalise previously acquired skills). This may have been a good addition to the training to assist Makena to generalise her ability to run intraverbal training across a wider range of activities. A second explanation could be that Dennis had a greater instant success in tacting than he did in intraverbals. Whilst running intraverbal targets Makena could not always successfully prompt behaviour from Dennis despite following the strategies provided, she had more success during tact training as Dennis was more likely to repeat echoic prompts, which could subsequently be faded as Dennis displayed more independent tacts. Given that Dennis’ increased verbal behaviour would likely act as a reinforcer for Makena’s behaviour, the matching law predicts that Makena would distribute her behaviour to match the ratio of reinforcement for each behaviour (Myerson & Hale, 1984), which could have in turn led to the uneven distribution of strategy use observed in Makena’s data. This could be rectified in future research by asking for three shorter videos depicting each strategy separately or by allocating a certain time within each video for each strategy and reporting rate of each operant per minute of allocated time. Additionally, parents could be asked to self-evaluate each video prior to sending them in, as this would allow them to recognise for themselves components to add in following play sessions. Past telehealth studies have indicated this is a useful addition to training (Meadan & Daczewitz, 2015; Vismara et al., 2013).

A second limitation to the study was the small number of participants. Despite the study using a multiple baseline across behaviours research design to achieve experimental control, replicating findings with only two participants allows for a weakened external validity which may in turn lead to inaccurate extrapolations to the population of reference. Future research should aim to replicate our findings with additional participants. The training received by the parents mediated the changes observed in the child communication outcomes. It would remain to be seen whether the changes observed in the verbal behaviour of the two children who took part in the project could be generalised to other participants. This may be especially questionable in the gains made in the tact variable. Both participants were motivated by certain play activities which were conducive to teaching tacting. Hugo enjoyed picture books, puzzles, and matching cards and Dennis liked playing with animal and cartoon figurines. Had these interests not been present, as could be the case for many children with a diagnosis of ASD, then it is doubtful whether the gains observed in the tacting variable would be observed. Future researchers may wish to conduct preference assessments prior to completing training to assess the compatibility of the play materials and the strategies taught, and condition neutral stimuli as reinforcers when necessary (Cló & Dounavi, 2020).

As in previous studies, there were some technical issues relating to the strength of Wi-Fi signal leading to a degradation in picture quality; these could be rectified with simple adjustments in location or device used. Scheduling of meetings was done with awareness of small time zone differences. Future research may consider using only asynchronous coaching with more detailed delayed feedback, should the time difference between countries be inconvenient, as has been demonstrated with professional interventionists (Neely et al., 2020). An additional limitation is present in the omission of fidelity data being collected on the trainer’s coaching of parents. Such fidelity data would have provided an indication of consistency in coaching across all training sessions and between participants, thus increasing both the internal and external validity of results. Future research should focus on collecting such fidelity data.

To sum, the study successfully demonstrated the use of a telehealth platform to train parents in naturalistic strategies which aimed to increase the verbal behaviour of children with ASD. This training produced positive outcomes in terms of increases in parental fidelity and child verbal behaviour for both families. The use of the telehealth platform allowed the parents to avail of services which would not have been accessible in the country/area of residence and enabled the trainer to provide services directly into the homes of families. Both parents rated the training and outcomes very favourably and would be likely to utilise telehealth training again. They felt the addition of coaching boosted their skills in a way their didactic training alone had not. The study took place under unprecedented conditions and provided a demonstration of the practicality of using a telehealth platform in times of international crisis.

## Supplementary Information

Below is the link to the electronic supplementary material.

Supplementary file1 (DOCX 19 KB)

## Declarations

### Research Involving Human and Animals

All procedures performed in studies involving human participants were in accordance with the ethical standards of the institutional research ethics committee and with the 1964 Helsinki declaration and its later amendments or comparable ethical standards.
