# Correction to: The Efficacy of Using Telehealth to Coach Parents of Children with Autism Spectrum Disorder on How to Use Naturalistic Teaching to Increase Mands, Tacts and Intraverbals

**DOI:** 10.1007/s10882-022-09870-9

**Published:** 2022-09-09

**Authors:** Jenny Ferguson, Katerina Dounavi, Emma A. Craig

**Affiliations:** School of Social Sciences, Queen’s University of Belfast, Education & Social Work69-71 University Street, Belfast, BT7 1HL Northern Ireland

## Correction to: Journal of Developmental and Physical Disabilities 10.1007/s10882-022-09859-4

In the original published version of this article, the author noted several mistakes in the references list:

- American Psychiatric Association. (2013). *Diagnostic and statistical manual of mental disorders* (5^th^ ed.). 10.1176/appi.books.97808-90425-596
- Awasthi, S., Aravamudhan, S., Jagdish, A., Joshi, B., Mukherjee, P., Kalkivaya, R., Ali, R. S., Srivastava, S. N., & Edasserykkudy, S. (2021). Transitioning ABA services from in clinic to telehealth: Case study of an Indian organization’s response to COVID-19 lockdown. *Behavior Analysis in Practice, 14(4),* 893–912. 10.1007/s40617-021-00600-914

Volume number and pages from this article were missing and have now been added.

- Behavior Analyst Certification Board. (2018). RBT ^®^ Task List (2^nd^ ed.). https://www.bacb.com/wp-content/uploads/2020/05/RBT-2nd-Edition-Task-List_181214.pdf

This reference edition and link were missing and have now been added.

- Cló, E., & Dounavi, K. (2020). A systematic review of behaviour analytic processes and procedures for conditioning reinforcers among individuals with autism, developmental or intellectual disability. *European Journal of Behavior Analysis, 21*(2), 292–327. 10.1080/15021-149.2020.1847953
- Colombo, R. A., Wallace, M., & Taylor, R. (2020). An essential service decision model for ABA providers during crisis. *Behavior Analysis in Practice, 13(3)*, 306–311. 10.1007/s40617-020-00432-z

Volume and issue numbers and pages from these articles were missing and have now been added.

- Cooper, J., Heron, T., & Heward, W. (2019). *Applied behavior analysis* (3^rd^ ed.). Pearson. Hoboken, NJ: Pearson Education.

Publisher location was missing from this book and has now been added.

- Craig, E. A., Dounavi, K., & Ferguson, J. (2021). Telehealth to train interventionists teaching functional living skills to children with autism spectrum disorder. *Journal of Applied Behavior Analysis, 54*(2), 511–529. 10.1002/jaba.834

Doi was missing from this article and has now been added.

- Craig, E. A., Dounavi, K., & Ferguson, J. (in press). Effectiveness of a brief functional analysis and functional communication training conducted through telehealth. *Journal of Developmental and Physical Disabilities*. 10.1007/s10882-022-09857-6

The title of this reference was wrong and has now been corrected in line with 10.1007/s10882-022-09857-6

- Dounavi, K., & Dillenburger, K. (2013). Behaviour analysis and evidence-based education, *Effective Education,* *4*(2), 191–207. 10.1080/19415532.2013.855007

Volume and issue number and pages from this article were missing and have now been added.

- Ferguson, J., Craig, E. A., & Dounavi, K. (2019). Telehealth as a model for providing behaviour analytic interventions to individuals with Autism Spectrum Disorder: A systematic review. *Journal of Autism and Developmental Disorders, 49*(2), 582–616. 10.1007/s10803-018-3724-5

Page numbers were missing from this article and have now been added.

- Gevarter, C., Najar, A. M., Flake, J., Tapia-Alvidrez, F., & Lucero, A. (2022). Naturalistic communication training for early intervention providers and Latinx parents of children with signs of autism. *Journal of Developmental and Physical Disabilities, 34*(1), 147–169. 10.1007/s10882-021-09794-w

This reference has been updated to reflect the inclusion of this paper in a volume. In-text citation has also been updated.

- Hume, K., Steinbrenner, J. R., Odom, S. L., Morin, K. L., Nowell, S. W., Tomaszewski, B., Szendrey, S., McIntyre, N. S., Yücesoy-Özkan, S., & Savage, M. N. (2021). Evidence-based practices for children, youth, and young adults with autism: Third generation review. *Journal of Autism and Developmental Disorders, 51*(11), 4013–4032. 10.1007/s10803-020-04844-2
- Lord, C., Rutter, M., DiLavore, P. C., Risi, S., Gotham, K., & Bishop, S. (2012). *Autism diagnostic observation schedule* (2^nd^ ed.). Torrance, CA: Western Psychological Services.

Publisher information was missing from this reference and has now been added.

- McGarry, E., Vernon, T., & Baktha, A. (2019). Brief report: A pilot online pivotal response treatment training program for parents of toddlers with autism spectrum disorder. *Journal of Autism and Developmental Disorders, 50*(9), 3424–3431. 10.1007/s10803-019-04100-2
- Rodriguez, K. A. (2020). Maintaining treatment integrity in the face of crisis: A treatment selection model for transitioning direct ABA services to Telehealth. *Behavior Analysis in Practice, 13*(3), 291–298. 10.1007/s40617-020-00429-8

Volume number and pages from these articles were missing and have now been added.

- Pollard, J. S., LeBlanc, L. A., Griffin, C. A., & Baker, J. M. (2021). The effects of transition to technician-delivered telehealth ABA treatment during the COVID-19 crisis: A preliminary analysis. In *Journal of Applied Behavior Analysis 54*(1), 87–102. 10.1002/jaba.803
- Rutter, M., Le Couteur, A., & Lord, C. (2003). *Autism diagnostic interview, revised.* Los Angeles, CA: Western Psychological Services.

Publisher information was missing from this reference and has now been added.

- Skinner, B. F. (1957). *Verbal behavior.* New York: Appleton-Century-Crofts.

Publisher location was missing; reference has now been corrected.

- Wong, C., Odom, S. L., Hume, K., Cox, A. W., Fettig, A., Kucharczyk, S., Brock, M. E., Plavnick, J. B., Fleury, V. P., & Schultz, T. R. (2014). Evidence-based practices for children, youth, and young adults with Autism Spectrum Disorder. *Journal of Autism and Developmental Disorders, 45(7)*, 1951–1966. 10.1007/s10803-014-2351-z

Journal name has been corrected for this paper and volume and page numbers have been added.

- World Health Organization. (2021). Leveraging telehealth for efficient delivery of primary health care in the WHO South-East Asia Region. https://apps.who.int/iris/bitstream/handle/10665/350199/Telehealth-PHC-eng.pdf?sequence=1&isAllowed=y

Reference title was incomplete and link obsolete.

This erratum is presented to fix these errors.

The original article has been corrected.

