# Supplementary material for: The Efficacy of Using Telehealth to Coach Parents of Children with Autism Spectrum Disorder on How to Use Naturalistic Teaching to Increase Mands, Tacts and Intraverbals
Source: J Dev Phys Disabil. 2022 Jul 29;35(3):417–47. doi: 10.1007/s10882-022-09859-4 (PMC9334541; doi:10.1007/s10882-022-09859-4)
Supplement: Supplementary file 1 — Supplementary file1 (DOCX 19 KB) [file 10882_2022_9859_MOESM1_ESM.docx]

**Supplementary Table 1**

*Fidelity check list for “Follow the Lead” strategy*

| Strategy Step | Operational definition | Recording method |
| --- | --- | --- |
| 1. Play area is set up with the child’s favourite items. | Play area should contain at least 5 potentially preferred items. | Partial |
| 2. Play shaped by motivation | Parents should wait for the child to initiate the play with any item. Initiation can include reaching for toy, looking towards parent to gain more of something (bubbles, tickles etc.). This can be scored if motivation has continued over from previous intervals or if new discrete episodes of child initiation are observed. If above behaviour is the result of a First-Then contingency to gain access to a different preferred activity, it will not be scored as motivation. | Partial |
| 3. Offers a choice (If applicable) | If the child is not engaging with any toys, preferred items can be placed in front of the child or offered, by demonstrating the fun properties of the item. | Partial |
| 4. Position is facing the child | Parents should position themselves facing the child unless the play does not allow for this, e.g. spinning etc. | Whole |
| 5. Joined in play appropriately. | Play should be joined in by adding on preferred items, no demands and demonstrating fun ways to play with items. Parents should not take over the play but should aim to follow their children’s lead. Prompting for verbal operants are not considered demands. | Whole |
| 6. Used language appropriately | Language should not involve demands and should be clear, concise and simple. | Whole |
| 7. Reinforces desirable behaviour. | Any desirable behaviours such as vocalisations, eye contact, imitation should be reinforced by praise (e.g. nice looking, Wow you copied!) and the item or activity if appropriate or a natural continuation of the activity that would be reinforcing in itself (e.g. child looks at Mum and she nods her head, smiles and presents a fun play action). | Partial |

*Note.* Reprinted from “The impact of a telehealth platform on ABA-based parent training targeting social communication in children with autism spectrum disorder” by Ferguson, Dounavi and Craig, 2022, *Journal of Developmental and Physical Disabilities* (<https://doi.org/10.1007/s10882-022-09839-8>) Copyright 2022 Springer Nature.

**Supplementary Table 2**

*Fidelity check list for “Follow the Lead” strategy*

*Descriptions and examples of each motivation creation strategy*

| Motivation Creation Strategy | Description | Examples |
| --- | --- | --- |
| Add-on | Parents provide their child with objects or activities which can enhance their current play. They showed the objects in your hand or did something exciting with them and waited for their child to indicate that they would want them. | Playing with blocks, you can provide additional blocks needed to play by taking them out of a bag one by one and holding them out for your child.   In a colouring activity you could provide additional crayons needed to colour in the picture. |
| Small amounts | Parents will provide a very small amount of the object or activity and wait for eye contact before providing access to more. | Whilst playing with Playdoh, you can give your child a small piece to play with, in order to created motivation for them to want more.  During bubbles, blow a few bubbles only. |
| Pause-play | In this strategy parents start to play with their child and then suddenly stop and pause the play until they look and request. This works best with social games and activities, such as tickles, singing songs or blowing bubbles. | Providing tickles and suddenly stopping the game before starting again when eye contact and request is provided.  Dancing to a favourite song and pausing the music to wait for eye contact and request. |
| With-hold items | Here you will deliberately withhold access to certain items that you know are needed to either finish a task or to play with the item | Playing with a ball popper game, you start the game without the balls and wait for eye contact and requests before providing access to the balls.  Whilst completing a puzzle, you hold on to the last piece of the puzzle, or you keep hold of the crayons needed to colour a picture. |
| Need help | The environment is contrived in a way that often help is needed to gain access to preferred items. This help can be provided for locating an item that is out of reach of the child or for accessing an item that is kept items in a clear jars or ziplock bag. | Your child is really motivated to play with a ball, when you enter the playroom the ball is located up high on a shelf out of reach of the child. You wait for your child to look at you and request before providing access.    You are playing car ramps with your child and you place the cars for the game in a see-through container that your child cannot open. Open the container when you get eye contact and request. |

*Note.* Reprinted from “The impact of a telehealth platform on ABA-based parent training targeting social communication in children with autism spectrum disorder” by Ferguson, Dounavi and Craig, 2022, *Journal of Developmental and Physical Disabilities* (<https://doi.org/10.1007/s10882-022-09839-8>) Copyright 2022 Springer Nature.
